# Publisher Correction: Spermine synthase deficiency causes lysosomal dysfunction and oxidative stress in models of Snyder-Robinson syndrome

**DOI:** 10.1038/s41467-017-02462-8

**Published:** 2018-01-18

**Authors:** Chong Li, Jennifer M. Brazill, Sha Liu, Christofer Bello, Yi Zhu, Marie Morimoto, Lauren Cascio, Rini Pauly, Zoraida Diaz-Perez, May Christine V. Malicdan, Hongbo Wang, Luigi Boccuto, Charles E. Schwartz, William A. Gahl, Cornelius F. Boerkoel, R. Grace Zhai

**Affiliations:** 1 Department of Molecular and Cellular Pharmacology, University of Miami Miller School of Medicine, 33136 Miami, FL USA; 2 School of Pharmacy, Key Laboratory of Molecular Pharmacology and Drug Evaluation (Yantai University), Ministry of Education, Collaborative Innovation Center of Advanced Drug Delivery System and Biotech Drugs in Universities of Shandong, Yantai University, 264005 Yantai, Shandong China; 3 NIH Undiagnosed Diseases Program, National Human Genome Research Institute, NIH, 20892 Bethesda, MD USA; 4 Section of Human Biochemical Genetics, Medical Genetics Branch, National Human Genome Research Institute, NIH, 20892 Bethesda, MD USA; 5 JC Self Research Institute, Greenwood Genetic Center, 29646 Greenwood, SC USA; 6 Office of the Clinical Director, National Human Genome Research Institute, NIH, 20892 Bethesda, MD USA; 7 Present Address: Department of Medical Genetics, University of British Columbia, V6H 3N1 Vancouver, BC Canada

Correction to: *Nature Communications* 10.1038/s41467-017-01289-7, published online 02 November 2017

The originally published version of this Article contained errors in Figure 1. In panel c, the grey shading denoting evolutionary conservation and the arrowheads indicating amino acids affected in Snyder-Robinson syndrome were displaced relative to the sequence. These errors have now been corrected in both the PDF and HTML versions of the manuscript.

