# Supplementary material for: Spermine synthase deficiency causes lysosomal dysfunction and oxidative stress in models of Snyder-Robinson syndrome
Source: Nat Commun. 2017 Nov 2;8:1257. doi: 10.1038/s41467-017-01289-7 (PMC5668419; doi:10.1038/s41467-017-01289-7)
Supplement: Supplementary file 1 — Supplementary Information [file 41467_2017_1289_MOESM1_ESM.pdf]

Supplementary Figures

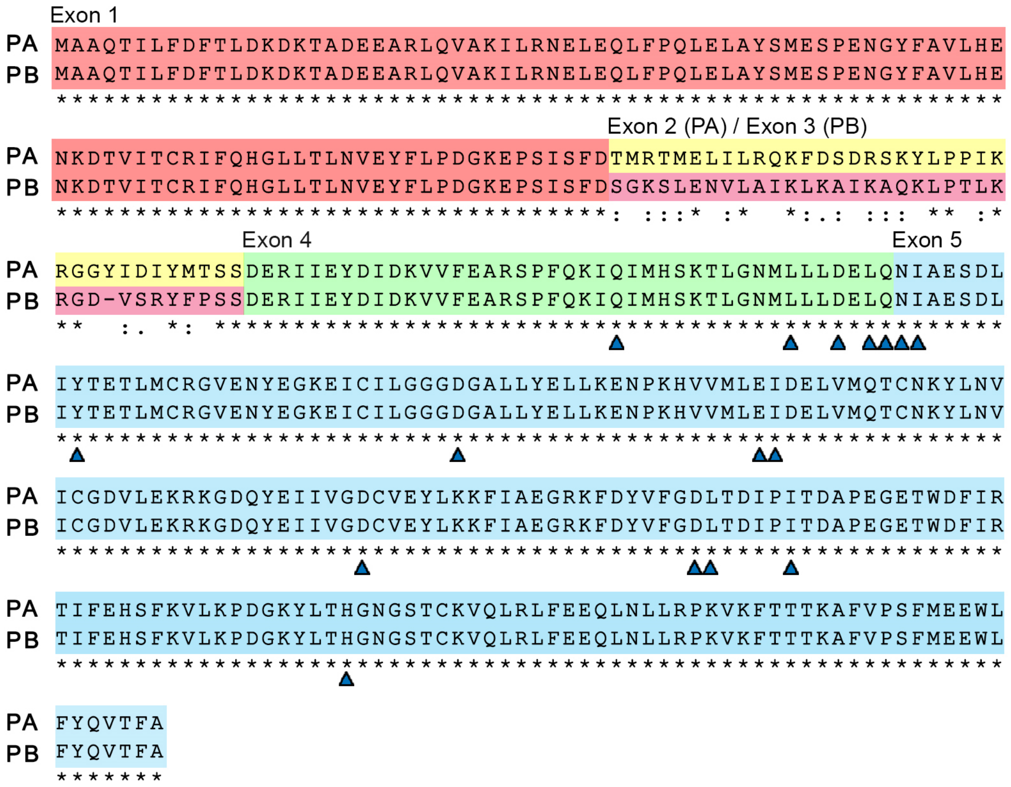

Supplementary Figure 1 Two predicted dSms isoforms share identical sites required for enzyme activity.

Protein sequence alignment of two isoforms of dSms, PA and PB. Exons coded with different colors. Blue arrowheads indicate required sites for enzyme activity. Asterisks mark the identical amino acid.

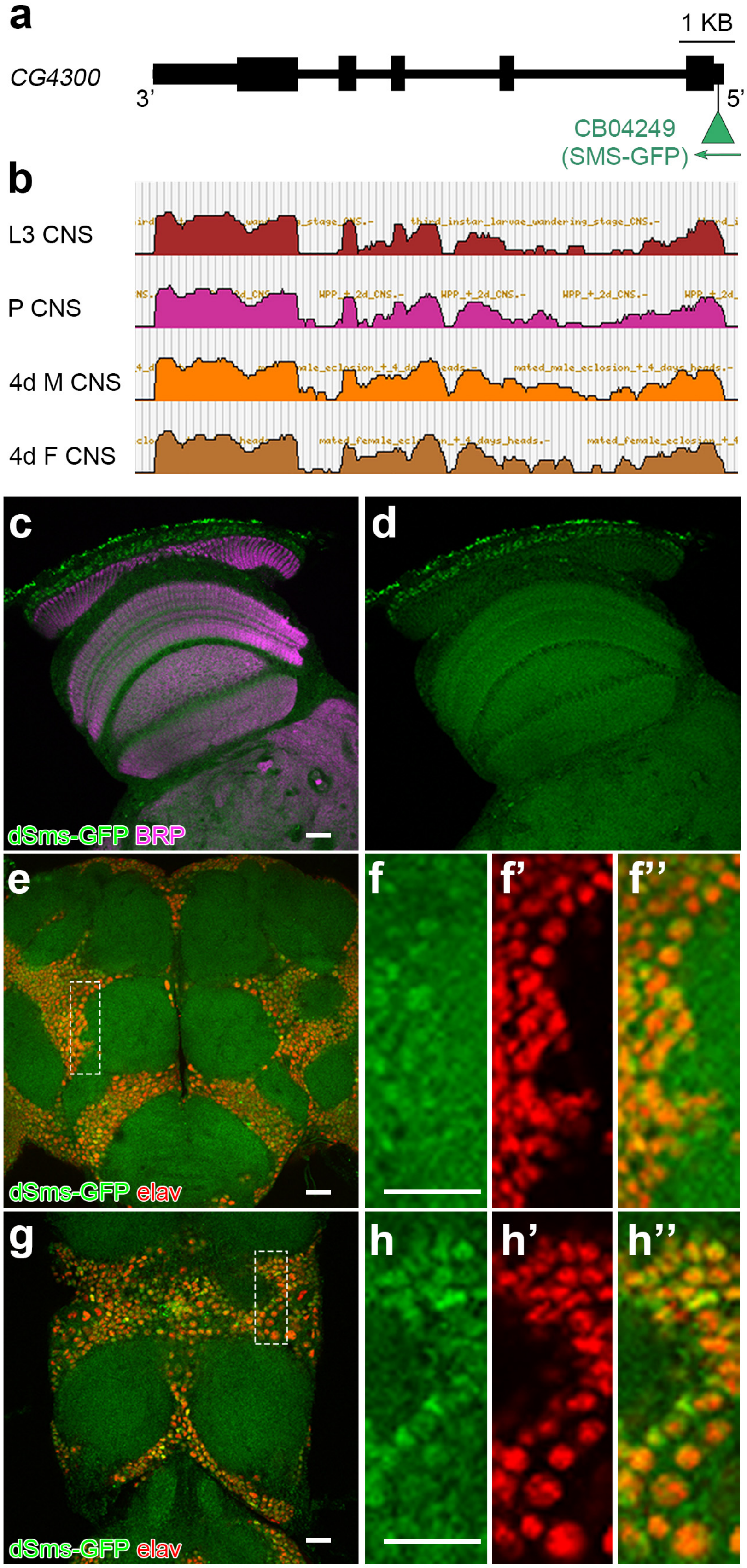

Supplementary Figure 2 dSms is expressed in *Drosophila* CNS.

(a) Genomic structure of GFP trap line *dSms-GFP*^{CB04249}. Arrow indicates the direction of the inserted P element.

(b) Expression profile of *dSms* at different developmental stages. L3, third instar larva; P, pupa; 4d M, 4 DAE male adult; 4d F, 4 DAE female adult. Signals are presented using log2 scaling method. Figure is generated from FlyBase: GBrowse Expression Levels (modENCODE).

(c-h") dSms is ubiquitously expressed in *Drosophila* optic lobe (**c, d**), middle brain (**e, f**), ventral nerve cord (**g, h**), and enriched in the synapses (colocalized with synaptic marker BRP; **c, d**) and neuronal cell bodies (colocalized with neuronal transcription factor elav; **f-f"**, **h-h"**). Scale bar, 20 μm.

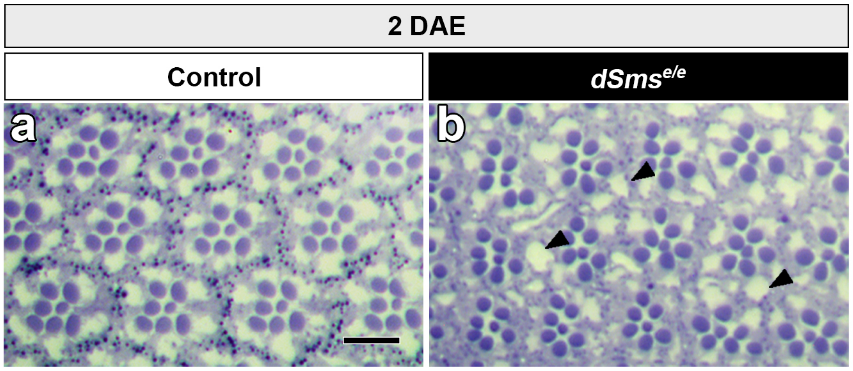

Supplementary Figure 3 Loss of dSms causes early-onset morphological abnormality of the ommatidia.

(**a, b**) Toluidine blue staining showing cross section of ommatidia of 2 DAE control (**a**) and *dSms*$^{e/e}$ (**b**) fly eyes. Black arrowheads indicate vacuoles. Scale bar, 5 µm.

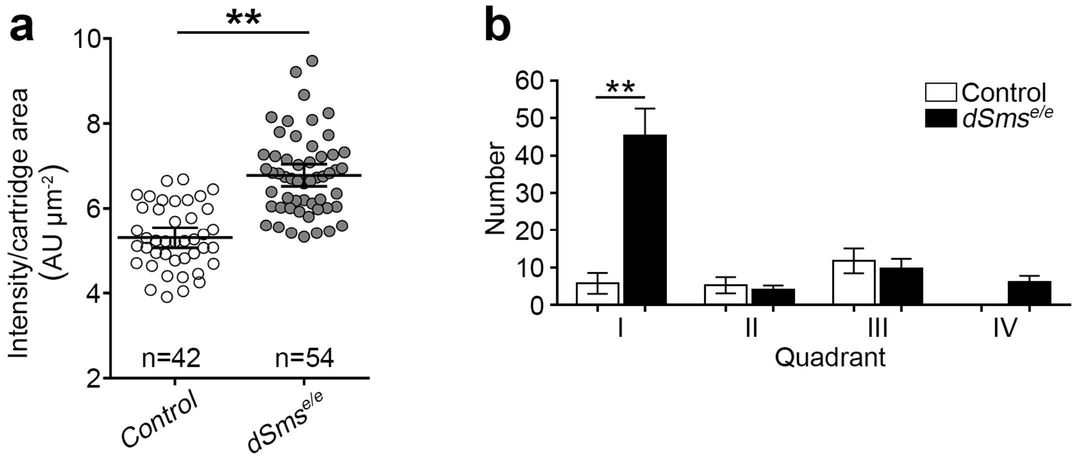

Supplementary Figure 4 Disruption of endocytosis and autophagy-lysosome flux in the synapses of *dSms*$^{e/e}$ flies.

(**a**) Scatter dot plot showing quantification of Rab5 intensity (mean ± 95% CI) in the cartridges of lamina synapses. Each data point represents the total intensity (AU) divided by the area of the cartridge (μm^2). n ≥ 42 cartridges obtained from 4 animals for each group.

(**b**) Quantification of Atg8a puncta (mean ± S.E.M.; control, n = 4; *dSms*$^{e/e}$, n = 3 animals) categorized into four quadrants. Increased Atg8a-positive puncta in quadrant I are detected in *dSms*$^{e/e}$ flies, suggesting the expansion of the autophagosome.

Student *t* test, $^{**}P < 0.01$.

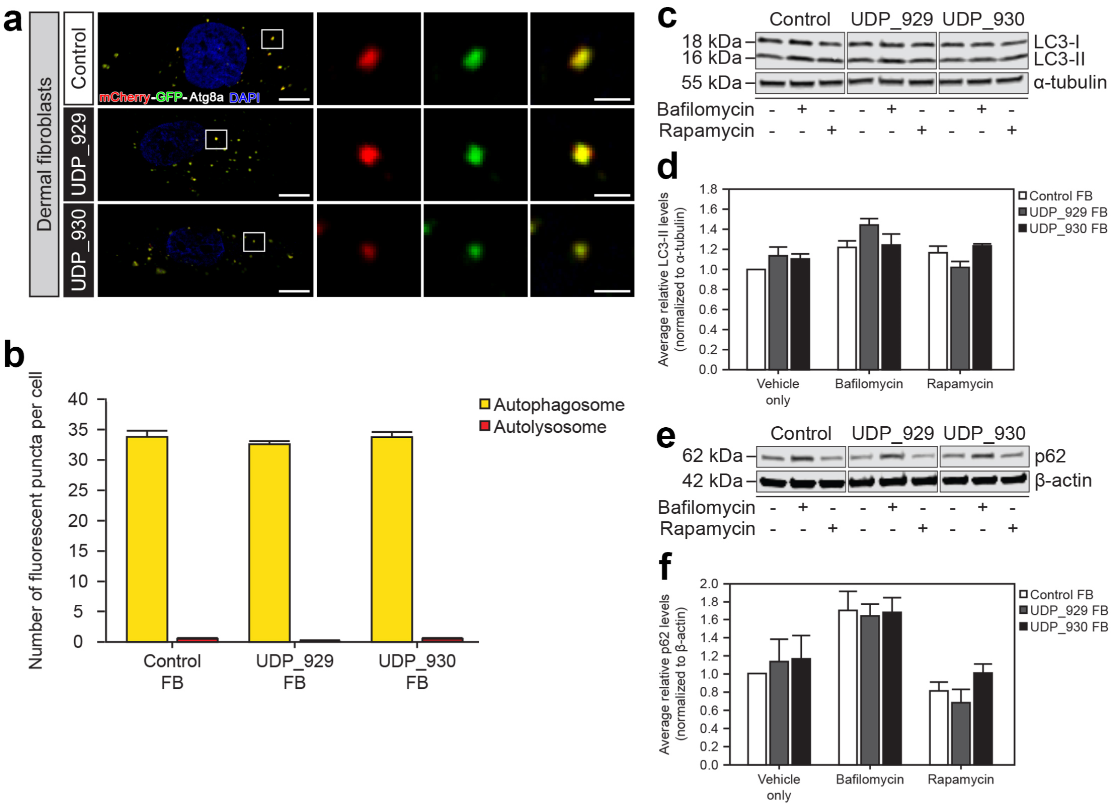

Supplementary Figure 5 Autophagic flux is not affected in SRS dermal fibroblasts.

(**a**) Confocal micrographs of LC3 flux examined through RFP-GFP-LC3 viral transduction in BMSCs. Boxed area (left) indicates magnified area (right) of autophagosomes and/or autolysosomes.

(**b**) Quantification (mean ± S.D.; n = 20 cells per sample) of the numbers of autophagosomes (RFP and GFP positive puncta) and autolysosomes (only RFP positive puncta).

(**c, e**) Western analysis of cell extracts from fibroblasts treated with or without autophagy inhibition (bafilomycin) or induction (rapamycin) with antibodies against LC3 (**c**), p62 (**e**) and β-actin.

(**d, f**) Quantification (mean ± S.D.; n = 3 extractions) of LC3-II (**d**) and p62 (**f**) levels normalized to β-actin normalized to untreated control fibroblasts.

Scale bars, whole cell image, 10 µm; magnified image, 2 µm.

d, **e**, One-way ANOVA post hoc Sidak (comparisons of preselected pairs) test; *$*P < 0.05$, $** P < 0.01$*.

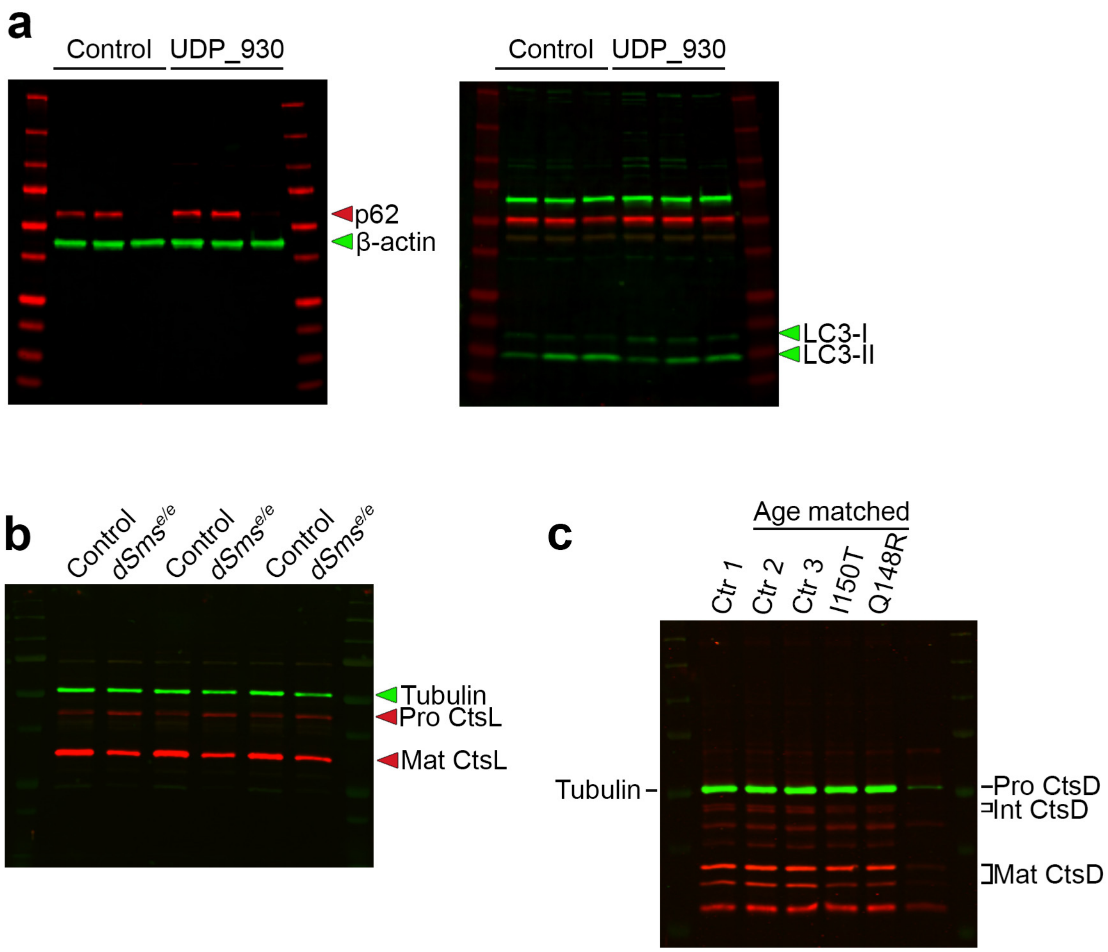

Supplementary Figure 6 Full size blot with size markers.

(**a**) Full size blot for main figure 4**c**. (**b**) Full size blot for main figure 5**e**. (**c**) Full size blot for main figure 5**h**.

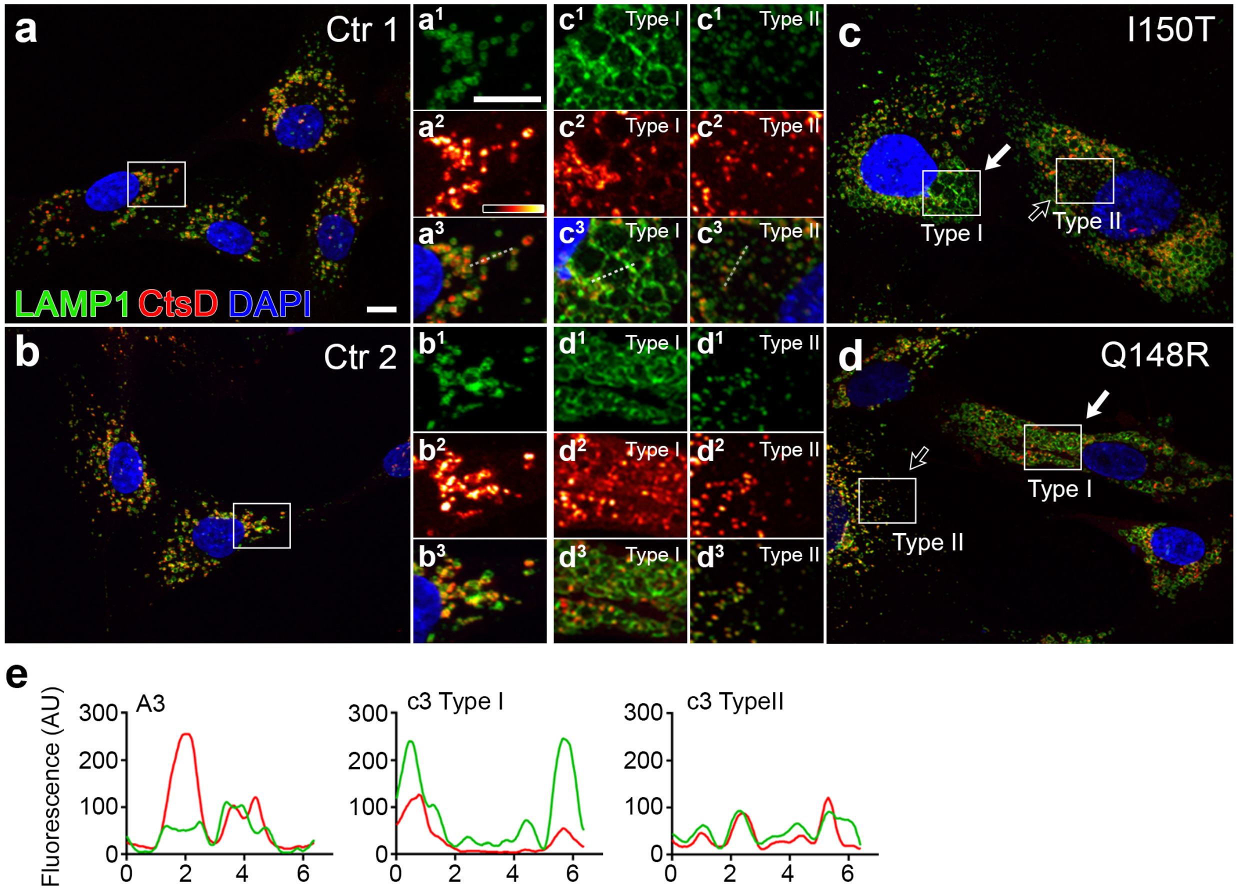

Supplementary Figure 7 Impaired lysosomal activity in SRS fibroblasts.

(**a-d**) Immunostaining of lysosomal membrane and lysosomal protease using markers LAMP1 and cathespin D (CtsD), respectively, on fibroblasts of age matched controls and SRS patients (altered amino acids indicated). Note enlarged (white arrow, Type I, high magnification $\mathbf{c^1}$-$\mathbf{d^3}$) and fragmented (black arrow, Type II, high magnification $\mathbf{c^1}$-$\mathbf{d^3}$) LAMP1 pattern presented in both SRS patient fibroblasts. Scale bar, 10 µm.

(e) Histogram plots of the fluorescence intensity of LAMP1 (green) and cathepsin D (red) in arbitrary units (AU) across a control lysosome versus SMS mutant lysosomal phenotypes I and II (along the dotted lines indicated in $\mathbf{a^3}$ and $\mathbf{c^3}$).

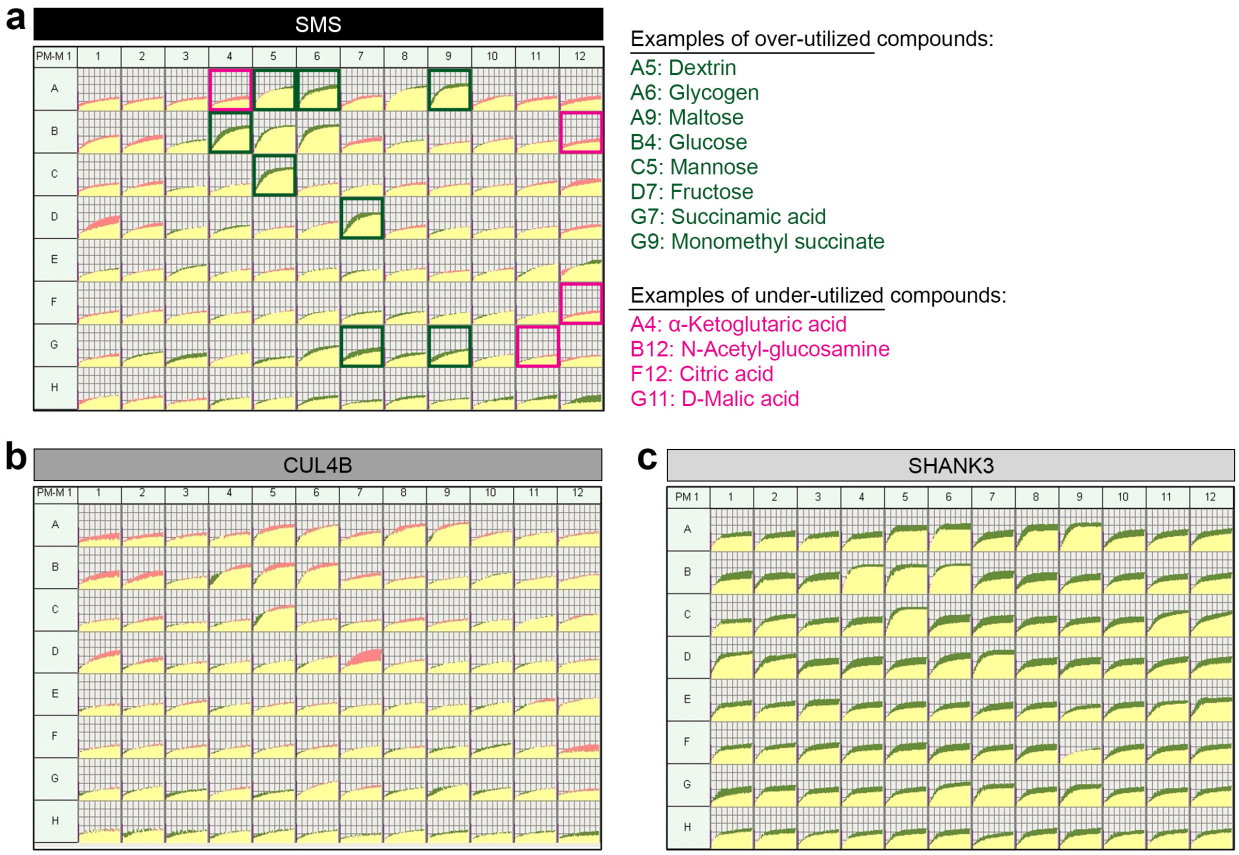

Supplementary Figure 8 Metabolic profile of SRS lymphoblastoid cells.

(a) Left, representative Phenotype Microarray plate view showing metabolic profile of lymphoblastoid cells from one SRS patient. Curves from each well are generated by comparing respiration kinetics from mutant cells to control and the difference is indicated in pink (under-utilized compounds) or green (over-utilized compounds). Right, list of compounds contained in the wells outlined in the plate. n = 14 SRS patients (unrelated) and n = 12 controls.

(b, c), Representative Phenotype Microarray plate view showing metabolic profile of CUL4B (**b**) and SHANK3 mutant lymphoblastoid cells (**c**).

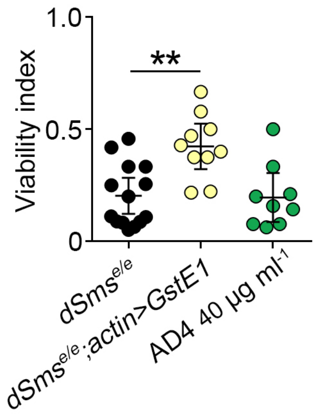

Supplementary Figure 9 Decreased viability of dSms mutant flies can be rescued through *GstE1* overexpression, but not feeding with AD4.

Scatter dot plot showing survival index (mean ± 95% CI) of *dSms*$^{e/e}$ flies with genetic modification and drug supplementation. Each data point represents a sample of ≥ 80 embryos.

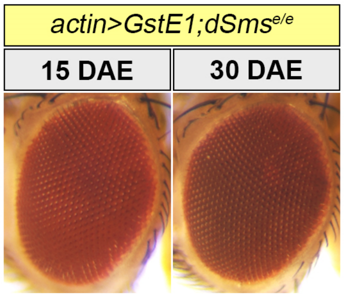

Supplementary Figure 10 Age-dependent pigmentation loss in dSms mutant flies can be rescued through GstE1 overexpression.

Eye exterior morphology of flies at different ages.

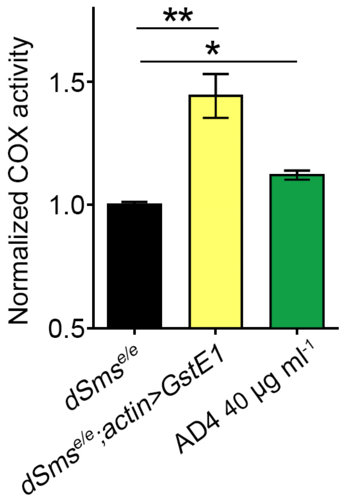

Supplementary Figure 11 Decreased COX activity in dSms mutants can be restored through *GstE1* overexpression and AD4 feeding.

Quantification of COX activity in flight muscles of 5 DAE flies normalized to *dSms*$^{e/e}$ mutants. One-way ANOVA post hoc Tukey test; **P < 0.05, ** P < 0.01*.

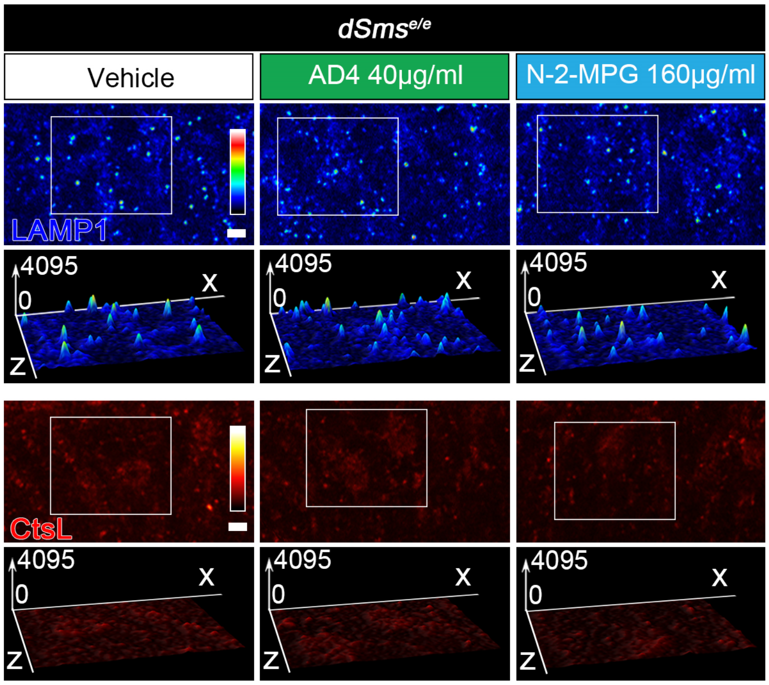

Supplementary Figure 12 Lysosomal integrity is not rescued through antioxidants supplementation.

Immunostaining of fly laminae labeled with LAMP1 for lysosome membrane and cathepsin L for lysosomal protease. The fluorescence intensity profile is illustrated in a three dimensional surface plot.
